# Ex Vivo High-Resolution Magic Angle Spinning (HRMAS) ^1^H NMR Spectroscopy for Early Prostate Cancer Detection

**DOI:** 10.3390/cancers14092162

**Published:** 2022-04-26

**Authors:** Annabel Steiner, Stefan Andreas Schmidt, Cara Sophie Fellmann, Johannes Nowak, Chin-Lee Wu, Adam Scott Feldman, Meinrad Beer, Leo L. Cheng

**Affiliations:** 1 Department of Pathology, Harvard Medical School/Massachusetts General Hospital, Boston, MA 02114, USA; cara-sophie@arcor.de (C.S.F.); cwu2@mgh.harvard.edu (C.-L.W.); 2 Department of Diagnostic and Interventional Radiology, University Hospital Ulm, 89081 Ulm, Germany; stefan.schmidt@uniklinik-ulm.de (S.A.S.); meinrad.beer@uniklinik-ulm.de (M.B.); 3 Radiology Gotha, SRH Poliklinik Gera, 99867 Gotha, Germany; johannes.nowak@yahoo.de; 4 SRH University of Applied Health Sciences, 07548 Gotha, Germany; 5 Department of Urology, Harvard Medical School/Massachusetts General Hospital, Boston, MA 02114, USA; afeldman@partners.org; 6 i2SouI—Innovative Imaging in Surgical Oncology Ulm, University Hospital Ulm, 89081 Ulm, Germany; 7 Center for Translational Imaging “From Molecule to Man” (MoMan), University Hospital Ulm, 89081 Ulm, Germany; 8 Comprehensive Cancer Center Ulm (CCCU), University Hospital Ulm, 89081 Ulm, Germany; 9 Departments of Pathology and Radiology, Harvard Medical School/Massachusetts General Hospital, Boston, MA 02114, USA; lcheng@mgh.harvard.edu

**Keywords:** prostate cancer, NMR spectroscopy, metabolomics

## Abstract

**Simple Summary:**

Prostate cancer is the second leading cancer diagnosed in men worldwide. Current diagnostic standards lack sufficient reliability in detecting and characterizing prostate cancer. Due to the cancer’s multifocality, prostate biopsies are associated with high numbers of false negatives. Whereas several studies have already shown the potential of metabolomic information for PCa detection and characterization, in this study, we focused on evaluating its predictive power for future PCa diagnosis. In our study, metabolomic information differed substantially between histobenign patients based on their risk for receiving a future PCa diagnosis, making metabolomic information highly valuable for the individualization of active surveillance strategies.

**Abstract:**

The aim of our study was to assess ex vivo HRMAS (high-resolution magic angle spinning) ^1^H NMR spectroscopy as a diagnostic tool for early PCa detection by testing whether metabolomic alterations in prostate biopsy samples can predict future PCa diagnosis. In a primary prospective study (04/2006–10/2018), fresh biopsy samples of 351 prostate biopsy patients were NMR spectroscopically analyzed (Bruker 14.1 Tesla, Billerica, MA, USA) and histopathologically evaluated. Three groups of 16 patients were compared: group 1 and 2 represented patients whose NMR scanned biopsy was histobenign, but patients in group 1 were diagnosed with cancer before the end of the study period, whereas patients in group 2 remained histobenign. Group 3 included cancer patients. Single-metabolite concentrations and metabolomic profiles were not only able to separate histobenign and malignant prostate tissue but also to differentiate between samples of histobenign patients who received a PCa diagnosis in the following years and those who remained histobenign. Our results support the hypothesis that metabolomic alterations significantly precede histologically visible changes, making metabolomic information highly beneficial for early PCa detection. Thanks to its predictive power, metabolomic information can be very valuable for the individualization of PCa active surveillance strategies.

## 1. Introduction

Prostate cancer (PCa) is the second most frequently diagnosed cancer and the fifth most frequent cause of cancer-related deaths in men worldwide [1], thus representing a tremendous burden for public health systems. So far, the detailed etiology of PCa remains largely unexplained, with only a few established risk factors, including age, positive family history and ethnic origin [2].

At present, one of the most urging challenges in PCa diagnostics and therapy is the precise differentiation between patients with highly aggressive tumors and those with indolent tumors [3,4]. This distinction is essential for deciding on adequate, stage-adapted therapy strategies. Whereas patients with malignant tumors should immediately receive a curative therapy, those with indolent forms need protection from overtreatment with invasive therapies [3,4,5]. A transrectal ultrasound (TRUS)-guided systematic needle biopsy followed by histopathological evaluation is the current gold standard in PCa diagnostics [6]. Histopathological grading of prostate tumors, an important prognostic indicator, regularly follows the Gleason system [7,8].

The introduction of prostate-specific antigen (PSA) screening and an increase in prostate biopsy samples from 6 to 12 both resulted in a substantial rise in early-stage PCa diagnoses [9,10], indicating an essential need for higher diagnostic accuracy and more precise malignancy differentiation in early cancer detection [11]. Although PSA is used as a tumor marker and screening parameter, it is not tumor- but only prostate-specific [12] and, apart from intraindividual variations, can rise in the context of several circumstances other than PCa, such as benign prostatic hyperplasia, prostatitis or other manipulations of the prostate [12,13].

Additionally, with prostate tumors usually showing multifocal growth behavior [14], a significant number of cancer foci remains undetected during prostate biopsies, and tumor aggressiveness is often underestimated [14,15].

To summarize, current PCa diagnostic standards lack sufficient accuracy, as well as reliability, in distinguishing indolent from aggressive tumors. Therefore, in order to satisfy the requirement for personalized tumor- and stage-adapted therapy, more reliable screening strategies, diagnostic methods and biomarkers are needed [11].

HRMAS ^1^H NMR spectroscopy is among the diagnostic methods that have been investigated for this cause. It enables the ex vivo analysis of tissue samples with sufficient spectral resolution by spinning them at an angle of 54.7° away from the direction of the spectrometer’s static magnetic field [11,16]. Moreover, tissue structure preservation during NMR experiments allows for subsequent histopathological and genetic evaluation of samples [17,18] and, thus, the analysis of correlations between tissue metabolites and pathologies [18].

Therefore, metabolite quantification with ex vivo HRMAS ^1^H NMR spectroscopy represents a promising tool for investigating biochemical processes underlying PCa development and progression. The metabolome composition, meaning the entirety of all measurable metabolites [19,20], changes dynamically as the biological system reacts to genetic and environmental stimuli, such as diseases like cancer [19,20]. Specific metabolomic alterations are characteristic of malignant tumor cells [21], making the evaluation of cancer-specific metabolomic profiles diagnostically extremely valuable [11,22]. In several studies, metabolomic information acquired with ex vivo NMR spectroscopy has shown its potential for PCa detection, characterization and prognostic evaluation [22,23,24]. In this context, it was shown that the analysis of metabolomic profiles has superior accuracy compared to that of single metabolites [11,24].

Several authors suggested that metabolomic alterations significantly precede histologically visible changes [11,25]. Consequently, metabolomic information might be highly beneficial for the analysis of early prostate cancer development and behavior [11]. However, to our knowledge, no work exists that has examined individual metabolite concentrations and metabolomic profiles in histobenign samples and correlated them with later evolution (development of carcinoma vs. persistently benign).

Based on this assumption, the purpose of our study was to evaluate the diagnostic value of ex vivo NMR spectroscopy for early PCa detection by correlating metabolomic information with histopathology. In particular, we focused on assessing the predictive potential of metabolomic alterations in prostate biopsy samples of histobenign patients for a prostate cancer diagnosis in the following years. The aim was to answer the question of whether metabolomic data can separate a group of histobenign patients into two subgroups according to their risk for a future malignant transformation. A further object of this study was the differentiation of Gleason score (GS) categories 3 + 3 = 6 and 3 + 4 = 7 based on metabolite concentrations. Moreover, we wanted to evaluate whether there are linear correlations between metabolite intensities/metabolomic profiles and the PSA density (PSAd) as well as the volume percentage of benign epithelium in the tissue sample (Vol.%Epi).

## 2. Materials and Methods

### 2.1. Patients

This study is part of a primary prospective study. Before its start in 2006, an independent ethics committee, the Partners Human Research Committee Institutional Review Board, reviewed and approved the study (Protocol #: 2005P000892), and it was conducted according to specified rules and guidelines. Patients who underwent a prostate biopsy at the MGH Urology Department were considered for the study and only included after having given their written informed consent. From April 2006 until October 2018, 441 prostate tissue samples from 351 patients were progressively included in the study (90 patients participated with two prostate tissue samples each).

Clinical and pathological patient data were obtained from the Epic Partners patient database (Partners HealthCare International, Boston, MA, USA), including the following parameters: age at biopsy Bx0 (biopsy during which the NMR scanned sample(s) was/were taken), pre-Bx0 PSA, pre-Bx0 PSA density, prostate volume, American Joint Committee on Cancer Pathological Tumor Stage pTNM (in case of post-prostatectomy patients), GS of Bx0 (overall GS and GS of the NMR-analyzed sample(s)) and highest GS of all biopsies until the end of the study period.

**Figure 1:**
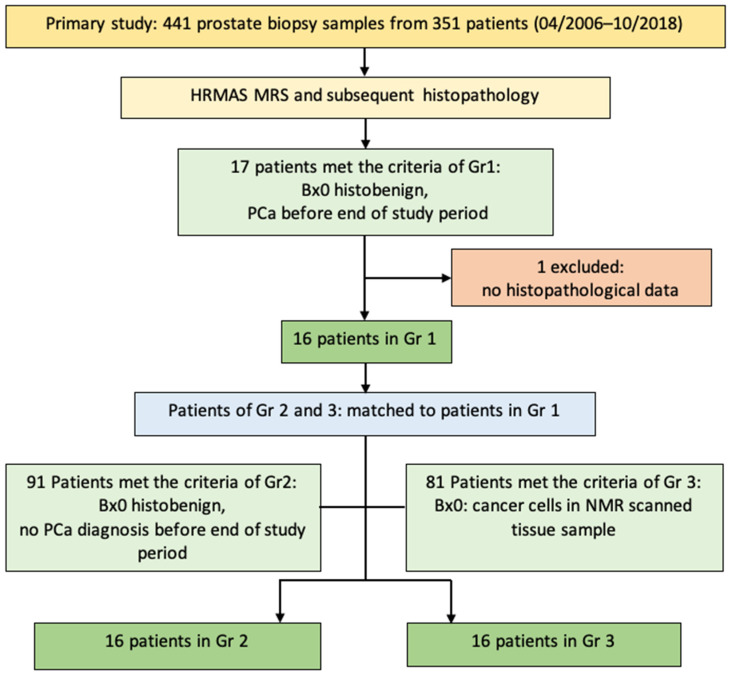
Flowchart of initial number of participants and inclusion and exclusion criteria. Abbreviations: Bx0 = biopsy during which the sample(s) for our study was/were taken, HRMAS MRS = high-resolution magic angle spinning nuclear magnetic resonance spectroscopy.

With regard to our research question, we performed a subgroup analysis of all 351 patients and built three homogenous groups of 16 patients (Gr): Gr1 and 2 included patients whose NMR scanned biopsy (Bx0) was histobenign, but Gr1 patients received a PCa diagnosis before the end of the study period, whereas Gr2 patients remained histobenign. NMR scanned biopsy samples of Gr3 already included cancer cells. The subgroup analysis included matching patients 1–16 of Gr2 and 3 to patients 1–16 of Gr1, following predefined clinical and histopathological matching criteria (pTNM for patients who underwent a prostatectomy, GS, PSAd, age at Bx0) (Figure 1). 

### 2.2. Intact Tissue Magnetic Resonance Spectroscopy (MRS)

During a patient’s biopsy, 1–2 additional tissue samples were taken for the study and analyzed with HRMAS ^1^H NMR spectroscopy on a Bruker Avance 600 MHz (14.1 Tesla) spectrometer (Bruker BioSpin Corp., Billerica, MA, USA) on the same day. Tissue cores were analyzed in a fresh, unfrozen state. Therefore, in order to prevent them from drying out and to minimize potential degradation of tissue metabolites, samples were placed in a construction of tubes functioning as a humidity chamber and stored on ice until the NMR experiment, as recommended by Tilgner et al. [26].

All spectrometer analyses were conducted according to the same protocol and without any knowledge of the clinical patient conditions. Tissue samples were placed into a 4 mm long rotor; then, 10 µL D_2_O (Sigma Aldrich, St. Louis, MO, USA) was added for field-locking. The spectra recording conditions were set as follows: temperature = 4 °C, repetition time = 5 s and the spectrometer resonance centered on the water resonance. A rotor-synchronized DANTE protocol with spinning rates of both 600 and 700 Hz for each sample was applied. 

HRMAS NMR data were first processed and analyzed with a lab-intern MatLab program (MathWorks, Natrick, MA, USA, Version 2009b). Integrals of spectral peaks in the range from 0.5 to 4.5 ppm (parts per million of magnetic field strength) were calculated using spectral curve fittings with Lorentzian–Gaussian line shapes and represented spectral peak intensities. Regions containing alcohol peaks and therefore indicating a potential contamination with biopsy gel were excluded from further analysis. The spectral range from 0.5 to 4.5 ppm was divided into 58 regions (Reg.), and spectral peak intensities were summed up to regional peak intensities (see Table A1 in Appendix A, which shows the spectral regions and their assigned ppm values). Regions were defined according to the spectral shape and a specific mathematical procedure with the aim of assigning whole peaks to one region and preventing peaks from being split up in between different regions. Spectral processing was verified in Acorn-NMR-Nuts (Livermore, CA, USA, 2D Professional version) in order to ensure the MATLab algorithm detected and integrated all peaks. Normalized spectral peak intensities were calculated for each region for better comparability of tissue samples within the study population. An outlier analysis of the spectral peak intensities was performed by calculating the Mahalanobis distance for each regional spectral peak intensity in SAS-JMP, with an upper control limit of 1.94. Metabolites and ppm values were assigned according to the literature (see Table A2 in Appendix B, which displays the assignment of metabolites to spectral regions), with peaks representing the signal of metabolites and peak intensities their concentrations. As regional peak intensities were compared instead of specific metabolites, it is possible that more than one metabolite contributed to the signal in a given region. Only those metabolites that, according to the literature, have a large contribution to a certain region and are likely associated with prostate cancer are further discussed in this study.

### 2.3. Quantitative Histopathology

After the HRMAS MRS analysis, tissue samples were histopathologically evaluated. First, they were fixed in 10% formalin and then embedded in paraffin. Afterwards, 5 µm sections were cut off the biopsy sample at 100 µm intervals throughout the sample and then stained with hematoxylin-eosin. Two genitourinary pathologists with considerable experience in the evaluation of prostate cancer tissue (18 and 9 years, respectively) conducted all histopathological analyses. They microscopically estimated the percentage area representing stroma, benign epithelium (incl. lumens) and cancerous tissue (incl. lumens) (rounded off to the nearest 5%). The volume percentage of each tissue type was calculated by multiplying the area percentage by the area size of the tissue slice. Moreover, each tissue slice containing cancer cells was also evaluated using the Gleason system. In accordance with the definition of our study groups, biopsy samples of 32 patients (Gr1 and 2) were histobenign, and those of 16 patients contained cancer cells. 

### 2.4. Statistical Analysis

Statistical tests were carried out in SAS JMP (Cary, NC, USA, Version JMP PRO 14) using the normalized spectral peak intensities calculated for 58 spectral regions and the first 12 principal components (PC). First, Shapiro–Wilks tests were performed to test for normal distribution. In order to analyze the data further, the following tests were performed: (1) analysis of variance (ANOVA) (normally distributed data) or Kruskal–Wallis–Wilcoxon test (non-normally distributed data) for the comparison of non-binary categorical variables (regional spectral peak intensities or PCs between all three groups); (2) student’s *t*-test (normally distributed data) or Mann–Whitney–Wilcoxon test (non-normally distributed data) for the comparison of binary categories (regional peak intensities and PCs between two groups, Gleason Score categories GS 3 + 3 = 6 and GS 3 + 4 = 7); (3) matched-pair analysis using a T test for paired data (normally distributed data) or the Wilcoxon signed-rank test (non-normally distributed data) for the comparison of regional spectral peak intensities between the matched pairs of two groups; (4) linear regressions of regional spectral peak intensities against the continuous variables Vol.%Epi and PSAd. Additionally, a multivariate analysis of covariance (MANCOVA) was performed in SPSS (IBM SPSS Statistics, Version 26, Armonk, NY, USA) in order to evaluate the effect of the variables age, PSAd and Vol.%Epi on the spectral peak intensities. The two-sided significance level for all statistical tests was set to α = 0.05. 

## 3. Results

### 3.1. Clinical and (Histo)Pathological Patient Data

Overall, prostate biopsy samples of 48 patients (one sample each) were evaluated NMR spectroscopically and histopathologically in this study. Baseline characteristics are listed in Table 1, with further patient data in Table 2 and results from the histopathological evaluation in Table 3. 

### 3.2. Differences between Histobenign and Malignant Prostate Tissue 

The following paragraphs only address the most relevant results. All significant results and *p*-values can be found in Table A3, Table A4, Table A5, Table A6 and Table A7 in Appendix C.

Peak intensities of several spectral regions were able to significantly differentiate between Gr2 and 3 and therefore histobenign and malignant prostate tissue, such as Reg. 23 (3.05–3.08 ppm; *p* = 0.0052), the peak intensity of which typically contains the signal of polyamines. Moreover, a principal component named PC 6 was also able to separate Gr2 and 3 (*p* = 0.0332). Therefore, in addition to single metabolites, a metabolomic profile was able to distinguish between histobenign and malignant prostate tissue. 

### 3.3. Differences between Histobenign and Premalignant Prostate Tissue 

**Figure 2:**
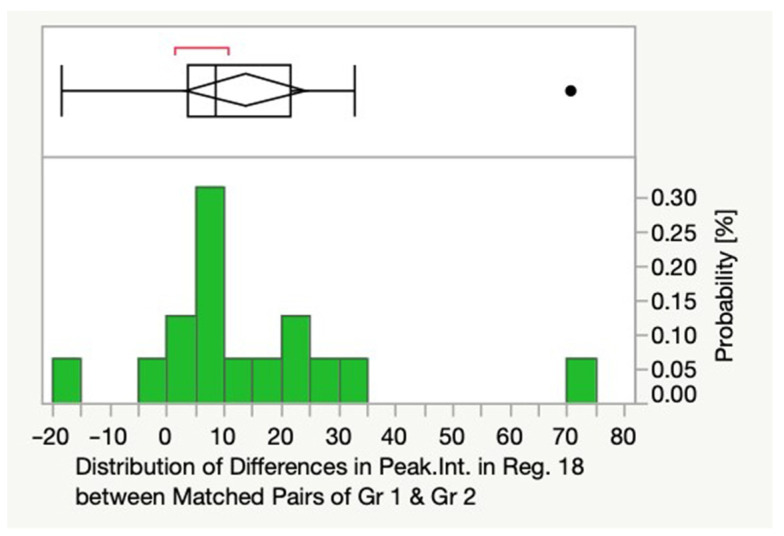
Distribution (boxplot above and histogram below) of differences in peak intensities in Reg. 18 (3.30–3.35 ppm) between matched pairs of Gr1 and Gr2. Abbreviations: Gr = group, Peak.Int = peak intensities, ppm = parts per million, Reg. = region. Length of the box = difference between the 25th and 75th percentiles; vertical line in the box = median of the data; whiskers (lines that extend from the box) = expected data variation (they extend 1.5 times the interquartile range from the left and the right side of the box); means diamond: top and bottom of the diamond are a 95% confidence interval for the mean, and the middle of the diamond is the sample average.

Peak intensities of several spectral regions and a principal component named PC 11 (*p* = 0.0365) were able to differentiate between Gr1 and 2 and therefore histobenign prostate tissue from patients who received a PCa diagnosis in the following years and those who remained histobenign. Reg. 18 (3.30–3.35 ppm; *p* = 0.0027) was one of these regions, with the signal of glycerophosphoethanolamine typically contributing to its peak intensity (Figure 2).

### 3.4. Differences between Premalignant and Malignant Prostate Tissue

Peak intensities of several spectral regions and a principal component called PC 1 (*p* = 0.0110) were able to distinguish between Gr1 and Gr3 and therefore premalignant and malignant prostate tissue. One example is Reg. 35 (2.30–2.38 ppm, *p* = 0.0092), which usually includes the signal of glutamate.

### 3.5. Differences between Gleason Score Categories GS 3 + 3 = 6 and 3 + 4 = 7

Peak intensities of several spectral regions were able to separate Gleason score categories GS 3 + 3 = 6 and 3 + 4 = 7, e.g., Reg. 27 (2.8–2.86 ppm; *p* = 0.0206), the peak intensity of which usually contains the signal of polyunsaturated fatty acid n-6 (PUFA n-6), and Reg. 23 (3.05–3.08 ppm; *p* = 0.0479), which usually represents the resonance of polyamines. This shows that metabolite intensities vary significantly according to Gleason score category.

### 3.6. Linear Correlations 

**Figure 3:**
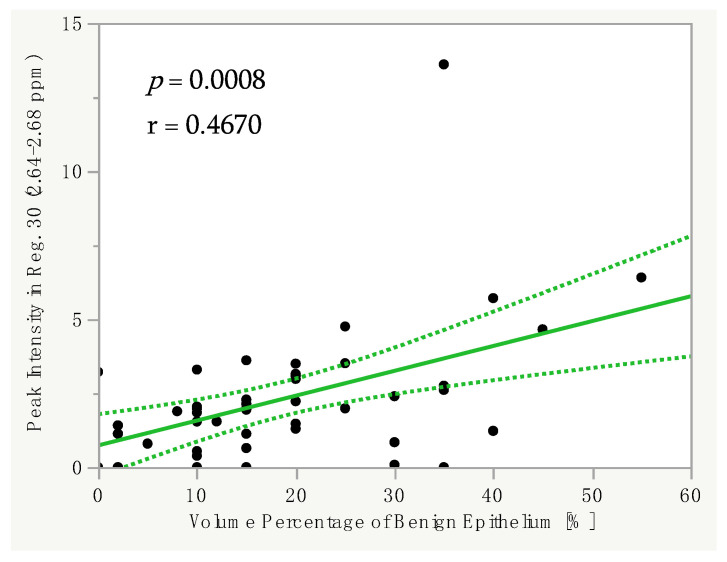
Linear correlation between the Vol.%Epi and the spectral peak intensity in Reg. 30 (2.64–2.68 ppm) in all groups. Dotted line = confidence interval. Abbreviations: Vol%Epi = volume percentage of benign epithelium, ppm = parts per million, Reg. = region.

The volume percentage of benign epithelium correlated significantly with the spectral peak intensities in several regions. For example, we found a significant positive linear correlation between the Vol.%Epi and the spectral peak intensity in Reg. 30 (2.64–2.68 ppm; *p* = 0.0008, r = 0.4670) in all groups (Figure 3). Typically, the signal of citrate largely contributes to the peak intensity in this region. Moreover, there was a significant positive linear correlation between the Vol.%Epi and the spectral peak intensity in Reg. 23 (3.05–3.08 ppm; *p* = 0.0399, r = 0.2976), which usually represents the signal of polyamines.

In Gr1, there was a significant positive linear correlation between the Vol.% Epi and Reg. 16 (3.63–3.65 ppm; *p* = 0.0095, r = 0.6257), with the signal of myo-inositol (MI) as a typical contributor to its peak intensity. In Gr1, there was also a significant negative linear correlation between the Vol.%Epi and the principal components P10 (*p* = 0.0304, r = −0.5411) and P11 (*p* = 0.0414, r = −0.5146).

**Figure 4:**
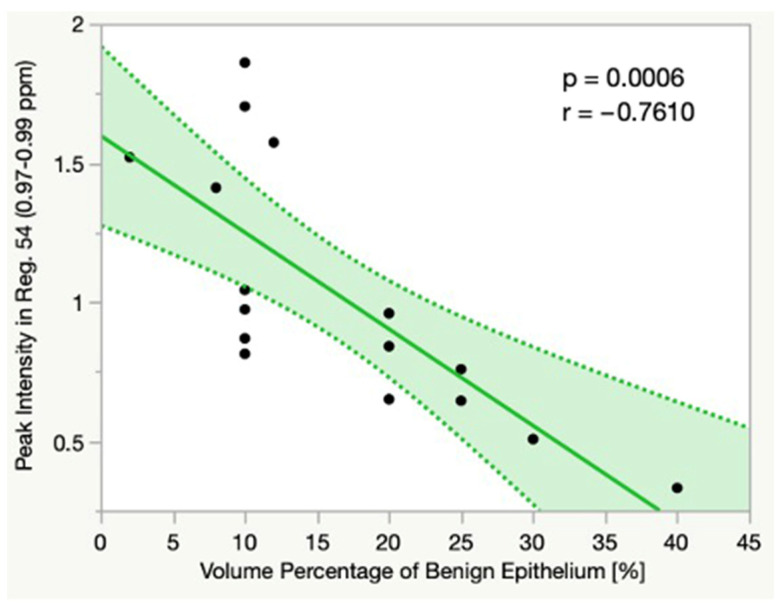
Linear correlation between the Vol.%Epi and the spectral peak intensity in Reg. 54 (0.97–0.99 ppm) in Gr3. Dotted line = confidence interval. Abbreviations: Vol%Epi = volume percentage of benign epithelium, ppm = parts per million, Reg. = region.

In Gr3, we found a significant negative linear correlation between the Vol.%Epi and the peak intensity in Reg. 54 (0.97–0.99 ppm; *p* = 0.0006, r = −0.7610), which usually contains the signals of isoleucine, leucine and valine (Figure 4).

**Figure 5:**
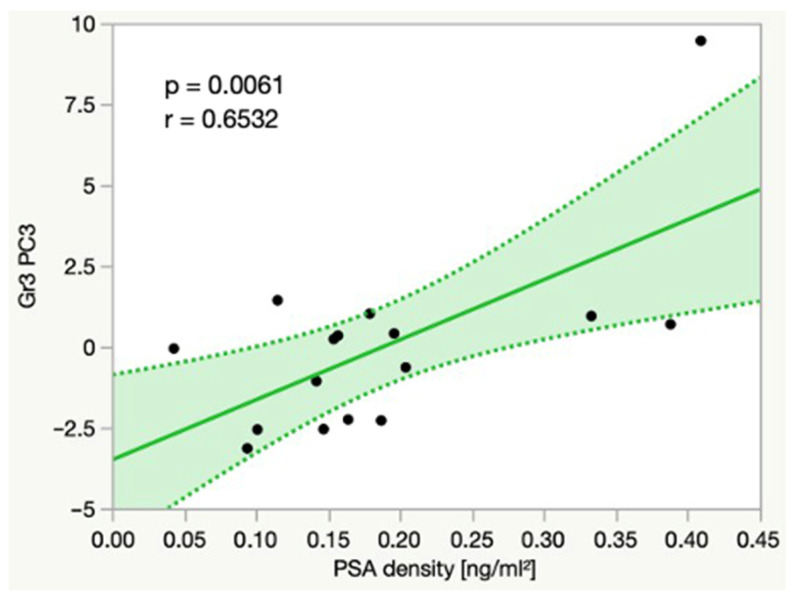
Linear correlation between the PSAd and a principal component called PC3 in Gr3. Dotted line = confidence interval. Abbreviations: Gr = group, ml = milliliters, ng = nanogram, PC = principal component, PSAd = PSA density.

Additionally, we were able to show significant linear correlations between the PSA density and multiple principal components, as well as the peak intensities of several spectral regions. For example, in Gr3, there was a significant positive linear correlation between the PSA density and Reg. 53 (1.00–1.06 ppm, *p* = 0.0047, r = 0.6682), which typically contains the signal of valine. In Gr3, the PSA density also significantly correlated with a principal component named PC3 (*p* = 0.0061, r = 0.6532) (Figure 5). 

## 4. Discussion

The major aim of our study was to evaluate metabolomic information as a biomarker for early PCa detection. However, in addition to confirming differences in metabolite intensities and metabolomic profiles between histobenign and malignant prostate tissue, we wanted to assess whether metabolomic information significantly differs between histobenign patients who received a PCa diagnosis before the end of the study period and those who remained histobenign. This predictive power of metabolomic alterations in prostate biopsy samples of histobenign patients could be very useful for the identification of patients at high risk of a future PCa diagnosis, as well as the individualization of active surveillance strategies based on a patient’s metabolomic risk profile.

### 4.1. Differentiation between Histobenign, Premalignant and Malignant Prostate Tissue

In our study, the peak intensity in Reg. 23, which usually has contributions from the signal of polyamines, was significantly different between Gr2 and Gr3. Consequently, the concentration of polyamines seems to vary significantly between histobenign and cancerous prostate tissue. Healthy prostatic epithelial cells produce and secrete high amounts of spermine, a function that is progrediently lost with malignant transformation [27]. Our results are in accordance with those of Swanson [28,29] who detected significantly higher polyamine values in histobenign compared to malignant prostate tissue with HRMAS MRS.

Furthermore, the peak intensity in Reg. 18 significantly differentiated between histobenign prostate tissue of patients who remained histobenign and of those who received a PCa diagnosis in the following years (Gr2 and Gr1). The signals of gylcerophosphoethanolamine (GPhE) and scyllo-inositol (SI) usually fall in this region. GPhE is involved in cell membrane metabolism, which is accelerated during carcinogenesis, resulting in higher GPhE concentrations [30]. Changes in the metabolism of SI and MI have also been discussed in this context [25,30,31]. According to our results, we can assume that the GPhE metabolism and the SI metabolism already change in a premalignant, histologically not yet visible stage of early prostate carcinogenesis. SI and GPhE concentrations measured with HRMAS MRS could thus function as indicators of early PCa development in histobenign prostate tissue. Swanson et al. [32] also investigated phosphoethanolamine metabolism in PCa with HRMAS MRS and found significantly higher GPhE/ethanolamine values in malignant compared to histobenign prostate tissue, although without evaluating changes in a histologically premalignant stage. Stenman et al. [31] were able to show significant negative correlations between the mean MI/SI ratio and tumor fraction, as well as tumor aggressiveness, indicating an increase in SI values with malignant transformation.

Reg. 35, which usually has contributions from glutamate, showed significantly different peak intensities between premalignant histobenign and malignant prostate tissue (Gr1 and Gr3). Glutamate levels tend to be high in PCa tissue due to increased glutaminolysis activity needed for tumor growth [27,33]. Our results imply that glutamate levels change as early malignant alterations progress towards the formation of a more advanced solid tumor. Madhu et al. [34] came to similar conclusions in their study; they detected that in comparison to histobenign prostate tissue, solely the glutamate levels of high-grade cancerous prostates showed a significant elevation, whereas those of low-grade PCa tissue were not significantly altered.

Unlike the authors of other studies, we were not able to show significant differences in citrate- and choline-containing metabolites between histobenign and malignant tissue. Why we were not able to observe these differences remains partly unclear; however, it could be due to the small sample size in each group (*n* = 16) and the low median GS, as measured differences also depend on the amount of cancer cells in the sample.

### 4.2. Correlations between Metabolite Intensities and Histopathology

We were able to show a significant positive linear correlation between the Vol.%Epi and the peak intensities in Reg. 23 and Reg. 30, usually containing the signals of polyamines and citrate. In addition to polyamines, prostatic epithelial cells also produce and secrete citrate into the prostatic fluid, which explains the changing of the citrate concentration with the amount of benign epithelium. This function is lost in PCa cells, which increasingly use citrate as a substrate for energy production [35]. These results are in accordance with those reported by Cheng [18] and Burns [36]. Differences in the concentrations of these two metabolites are thus not only due to malignant changes but also intraindividual variations in the amount of benign epithelium. In Gr3, the Vol.%Epi showed a significant negative correlation with the peak intensity in Reg.54, which usually has contributions from isoleucine, leucine and valine. So far, branched amino acids in prostate tissue have been rarely investigated, apart from one study, which detected decreased levels of branched amino acids in malignant cells [37], creating a need for further evaluation.

### 4.3. Correlations between Metabolite Intensities and PSA Density

In Gr3, the peak intensity in Reg. 53, usually including the signal of valine, significantly correlated with the PSAd, which led to the assumption that valine concentrations increase together with the PSAd. In contrast to our results, Dittrich et al. [38] found a significant linear negative correlation between the concentration of citrate and the PSAd. It is possible that the different composition of the study population accounted for the diverging results (PCa patients vs. histobenign patients).

### 4.4. Metabolite Concentrations for the Estimation of Tumor Aggressiveness

In our study, the peak intensity in Reg. 27, usually containing contributions from PUFAs n-6, was significantly different between biopsy samples of patients whose highest GS was 3 + 3 = 6 in the study period and of those with GS 3 + 4 = 7. Stenman et al. [39] investigated the PUFA metabolism of prostatectomy tissue with HRMAS MRS. PUFA n-6 could only be identified in malignant tissue with a GS of 3 + 4 = 7 but was undetectable in tissue with a GS of 3 + 3 = 6, which might explain our findings. Van Asten [40] was also able to differentiate malignancy degrees with HRMAS MRS, showing linear correlations between metabolite ratios and the GS. Consequently, measuring metabolites with HRMAS MRS could help to estimate tumor aggressiveness based on metabolic markers. 

Of particular note, the clinically important discrimination between Gleason scores 3 + 3 = 6 and 3 + 4 = 7 appears possible based on our results. Carcinomas with a score of 3 + 3 = 6 are considered low malignant, and carcinomas with a score of 3 + 4 = 7 are considered intermediate malignant. This has implications for further surveillance and therapy.

### 4.5. Metabolomic Profiles for Early PCa Detection

With the aim of detecting pathology-specific metabolomic profiles, a principal component analysis (PCA) was performed in our study, with the following results. PCs were able to significantly differentiate between histobenign (Gr2), premalignant (Gr1) and malignant prostate tissue (Gr3). This supports the assumption that there are combinations of metabolite concentrations, in the sense of a metabolomic profile, that significantly vary between histobenign, premalignant and malignant prostate tissue. Moreover, these metabolomic profiles seem to already be altered in a very early stage of malignant transformation, making the evaluation of metabolomic profiles extremely valuable for the identification of histobenign patients at high risk for a future cancer development. Furthermore, a PC each correlated with the Vol.%Epi (PC 10, *p* = 0.0304, r = −0.5411) in Gr1 and the PSAd in Gr3 (PC3, *p* = 0.0061, r = 0.6532), leading to the assumption that metabolomic profiles also vary according to histopathological features and the PSAd. Cheng et al. [11] were able to identify malignant tissue samples according to metabolomic profiles with an accuracy of 98.2%. Similarly to us, they found a significant linear correlation between one PC and the PSA value. Wu et al. [24] constructed a malignancy index based on metabolomic profiles acquired with HRMAS MRS, which was able to detect up to 97% of tumors. Giskeødegård et al. [41] classified benign and malignant prostate tissue samples according to their metabolomic profiles with a sensitivity of 86.9% and a specificity of 85.2%, and they were able to show significant linear correlations between metabolomic profiles and the volume percentage of benign glandular tissue, stroma and cancerous tissue, as well as Gleason score.

### 4.6. Limitations

A PCA results in completely independent factors and thus optimal results if the prerequisite of normal distribution is met. As our study also included non-parametric data, our results do not fulfill aforementioned optimality criterium. Furthermore, our study did not include a loading factor analysis for the identification of single metabolites contributing to a principal component, partly limiting the comparability with other studies. For facilitated comparability, an implementation of one-sided statistical hypothesis tests is necessary in future studies. Additionally, instead of investigating specific metabolites, we evaluated peak intensities in spectral regions. This allows for an explorative approach and the simultaneous analysis of the whole spectrum. However, as spectral regions can contain resonances from multiple metabolites, the peak intensities of regions discussed in this study might also have contributions from unmentioned metabolites. Moreover, our limited number of cancerous tissue samples (*n* = 16) and the low variance in Gleason score (2 categories) made it rather challenging to draw conclusions about malignant samples and malignancy degrees. Furthermore, with regards to predictive power, it is desirable to calculate exact positive and negative predictive values. However, in order to derive reliable predictive values, a larger sample size and a longer follow-up period than those implemented in our study would be required. This interesting calculation could be addressed in a future study.

## 5. Conclusions

In our study, we did not only confirm that metabolite intensities and metabolomic profiles are significantly different between histobenign and malignant prostate tissue. More importantly, we were able to show that metabolomic information can significantly differentiate between histobenign patients who are going to receive a prostate cancer diagnosis in the following years and those who can expect to remain histobenign. Our results are consistent with the hypothesis of other authors that the alteration of metabolomic prostate profiles starts in a premalignant histobenign stage. Therefore, metabolomic information could be very useful for early PCa detection, particularly due to its ability to identify histobenign patients at high risk of a future PCa diagnosis. This predictive power could help to individualize active surveillance strategies based on a patient’s metabolomic risk profile and improve PCa diagnostic and treatment strategies, thus contributing to a high level of personalized medicine. However, due to the lack of availability of high-resolution spectrometers and the high cost of such a procedure, implementation in routine diagnostics is rather challenging at present. Nevertheless, metabolomic information enhances fundamental understanding of early PCa development and could be used as a future diagnostic tool to inform early PCa diagnostics and supplement the current gold standard of histopathology.

## Figures and Tables

**Table 1:** Baseline characteristics at Bx0.

Clinical Parameter	Group	Mean	Standard Deviation	Minimum	Maximum	Unit
Age at Bx0	All Gr	62.29	7.23	44	77	years
	Gr1	60.25	6.28	46	71	
	Gr2	62.13	6.52	44	73	
	Gr3	64.50	8.49	46	77	
Pre-Bx0 PSA	All Gr	7.74	3.62	2.33	18.14	ng/mL
	Gr1	6.75	2.46	2.70	12.56	
	Gr2	8.84	3.43	3.50	18.14	
	Gr3	7.63	4.56	2.33	18.00	
Prostate Vol.	All Gr	46.58	28.84	18.14	182.00	mL
	Gr1	40.77	26.51	18.14	126.00	
	Gr2	57.00	39.35	24.00	182.00	
	Gr3	41.98	13.47	22.90	71.00	
PSAd	All Gr	0.20	0.11	0.04	0.50	ng/mL^2^
	Gr1	0.21	0.12	0.05	0.43	
	Gr2	0.19	0.11	0.06	0.50	
	Gr3	0.19	0.10	0.04	0.41	
**Biopsy characteristics**				**Number of Patients**		
Biopsy type:						
Fusion bx with 2 samples				14		
Regular bx with 1 sample				34		
Bx0 as 1st, 2nd or 3rd biopsy:						
1st				23		
2nd				13		
3rd				12		
Prostate region of Bx sample at regular biopsies:						
Right mid				27		
Right apex				1		
Right base				1		
No details provided				5		
Target region at fusion biopsies:						
Right target				4		
Left target				10		

Abbreviations: Bx = biopsy, Bx0 = biopsy during which the MRS scanned sample(s) was/were taken, mL = milliliter, MRS = magnetic resonance spectroscopy, ng = nanogram, Pat. = patient, PSA = prostate-specific antigen, PSAd = prostate-specific antigen density, Vol. = volume.

**Table 2:** Further clinical and pathological patient data.

Parameter	Number of Patients
Highest Bx GS until end of study period in Gr1 and Gr3:	
3 + 3 = 6	12
3 + 4 = 7	16
4 + 3 = 7	4
Pi-RADS all groups:	
2	1
3	5
4	4
5	5
Date of first PCa diagnosis in relation to date of Bx0 in Gr1:	
>2 y after Bx0 (Max: 5 y 6 m)	6
1–2 y after Bx0	6
<1 y after Bx0 (Min: 0 y 7 m)	4
Date of first PCa diagnosis in relation to date of Bx0 in Gr3	
At Bx0	12
<1 y before Bx0	1
1–2 y before Bx0	1
>2 y. before Bx0 (Max: 5 y 4 m)	2
Prostatectomy before end of study period in Gr1 and Gr3	
Yes	20
No	12
GS Prostatectomy	
3 + 3 = 6	2
3 + 4 = 7	13
4 + 3 = 7	4
4 + 5 = 9	1
Comparison of GS at Bx0 vs. GS at prostatectomy	
Same	10
Higher at PE	8
Higher at Bx0	2
pTNM	
T1c	1
T2a	1
T2c	5
T3a	12
N+	3
M+	3

Abbreviations: Bx = biopsy, Bx0 = biopsy during which the MRS scanned sample(s) was/were taken, GS = Gleason score, m = months, Max = maximal time, Min = minimal time, MRS = magnetic resonance spectroscopy, Pi-RADS = Prostate Imaging-Reporting and Data System, PCa = prostate cancer, pTNM = American Joint Committee on Cancer pathological tumor stage, y = years, m = months.

**Table 3:** Histopathological evaluation of MRS scanned biopsy cores.

Histopathological Parameter	Group	Mean	Standard Deviation	Minimum	Maximum	Unit
Vol.%Epi	All groups	18.77	12.36	0	55	%
	Gr1	21.38	16.72	0	55	
	Gr2	18.56	9.37	2	35	
	Gr3	16.38	9.91	2	40	
Vol.%Ca	Gr3	20.06	18.37	5	60	%
Vol.% Stroma	All groups	74.54	16.16	30	100	%
	Gr1	78.63	16.72	45	100	
	Gr2	81.44	9.37	65	98	
	Gr3	63.56	15.95	30	85	

Abbreviations: Bx0 = biopsy during which the MRS scanned sample(s) was/were taken, Gr = group, MRS = magnetic resonance spectroscopy, Vol.%Ca = volume percentage of cancer, Vol.%Epi = volume percentage of benign epithelium, Vol.%Stroma = volume percentage of stroma.

## Data Availability

The data presented in this study are available on request from the corresponding author. The data are not publicly available due to data privacy protection and the ongoing primary study.

## Appendix A

**Table A1:** Spectral regions 1 to 58 and their assigned ppm values.

Region	ppm Range	Region	ppm Range	Region	ppm Range
1	4.41–4.50	21	3.13–3.17	41	1.99–2.05
2	4.36–4.40	22	3.09–3.12	42	1.91–1.96
3	4.28–4.35	23	3.05–3.08	43	1.82–1.90
4	4.19–4.27	24	3.00–3.04	44	1.74–1.80
5	4.10–4.18	25	2.96–2.99	45	1.65–1.73
6	4.03–4.06	26	2.87–2.95	46	1.58–1.61
7	3.95–3.99	27	2.80–2.86	47	1.51–1.56
8	3.92–3.94	28	2.75–2.79	48	1.45–1.48
9	3.9–3.91	29	2.69–2.74	49	1.40–1.44
10	3.85–3.89	30	2.64–2.68	50	1.35–1.39
11	3.80–3.84	31	2.58–2.63	51	1.27–1.34
12	3.76–3.79	32	2.50–2.57	52	1.17–1.26
13	3.73–3.75	33	2.46–2.49	53	1.00–1.06
14	3.68–3.72	34	2.39–2.45	54	0.97–0.99
15	3.66–3.67	35	2.30–2.38	55	0.93–0.96
16	3.63–3.65	36	2.22–2.29	56	0.77–0.92
17	3.59–3.61	37	2.19–2.20	57	0.68–0.74
18	3.30–3.35	38	2.12–2.17	58	0.51–0.53
19	3.26–3.29	39	2.09–2.11		
20	3.20–3.25	40	2.06–2.08		

Spectra from prostate biopsy samples. Abbreviations: ppm = parts per million.

## Appendix B

**Table A2:** Assignment of metabolites to ppm values of chemical shift according to the literature. Govindaraju et al. measured chemical shifts of typical brain metabolites in solution. Mieckiwicz et al. measured chemical shifts of metabolites in serum samples, and the other listed authors below measured chemical shifts of metabolites in prostate tissue.

Regions	ppm Range	Metabolites	ppm Values	References
1	4.41–4.5			
2	4.36–4.4			
3	4.28–4.35	Phosphocholine	4.28	Govindaraju 2000
		ATP	4.295	Govindaraju 2000
4	4.19–4.27	Threonine	4.24	Govindaraju 2000
			4.26	Swindle 2008
5	4.1–4.18	Lactate	4.10	Govindaraju 2000
			4.10	Swindle 2008
			4.10–4.14	Jordan 2007
		Fructose	4.10–4.11	Mickiewicz 2014
		Proline	4.12	Mickiewicz 2014
6	4.03–4.06	Choline	4.05	Govindaraju 2000
		Tryptophan	4.05	Govindaraju 2000
7	3.95–3.99	Serine	3.97	Govindaraju 2000
		Phenylalanine	3.98	Govindaraju 2000
		Phosphoethanolamine	3.98	Govindaraju 2000
		Histidine	3.99	Govindaraju 2000
		Fructose	3.99	Mickiewicz 2014
8	3.92–3.94	Phosphocreatine	3.92	Govindaraju 2000
		Serine	3.93	Govindaraju 2000
		Tyrosine	3.93	Govindaraju 2000
		Creatine	3.94	Stenman 2011
9	3.9–3.91	Creatine	3.90	Swindle 2008
			3.91	Govindaraju 2000
10	3.85–3.89	Glucose	3.88	Govindaraju 2000
		Aspartate	3.89	Govindaraju 2000
		Fructose	3.89	Mickiewicz 2014
11	3.8–3.84	Fructose	3.82	Mickiewicz 2014
		Glucose	3.82–3.83	Govindaraju 2000
		Serine	3.83	Govindaraju 2000
12	3.76–3.79	Glutamine	3.76	Govindaraju 2000
		Alanine	3.76	Govindaraju 2000
			3.78	Stenman 2011
		Fructose	3.79	Mickiewicz 2014
13	3.73–3.75	Glutamate	3.74	Govindaraju 2000
		Glucose	3.75	Govindaraju 2000
14	3.68–3.72	Glycerophosphocholine	3.68	Zektzer 2005
			3.69	Swindle 2008
		Fructose	3.70	Mickiewicz 2014
		Glucose	3.70–3.71	Govindaraju 2000
15	3.66–3.67	Fructose	3.67	Mickiewicz 2014
Fructose	3.63–3.65	Phosphocholine	3.62	Govindaraju 2000
		Myo-inositol	3.63	Swanson 2006
17	3.59–3.61	Myo-inositol	3.51–3.61	Govindaraju 2000
			3.52–3.62	Stenman 2011
		Valine	3.60	Govindaraju 2000
		Phosphocholine	3.61	Swindle 2008
			3.62	Zektzer 2005
18	3.3–3.35	Glycerophosphoethanolamine	3.30	Swanson 2006
		Scyllo-inositol	3.30	Zektzer 2005
			3.33	Govindaraju 2000
			3.35	Stenman 2010, Stenman 2011
			3.35	Swanson 2006
19	3.26–3.29	Histidine	3.26	Govindaraju 2000
		Taurine	3.26	Swanson 2006
			3.26	Zektzer 2005
			3.28	Swindle 2008
		Myo-inositol	3.27	Govindaraju 2000
			3.28	Swanson 2006
			3.28	Zektzer 2005
			3.29	Stenman 2010, Stenman 2011
		Phenylalanine	3.28	Govindaraju 2000
20	3.2–3.25	Choline	3.19	Van Asten 2008
			3.20	Stenman 2010, Stenman 2011
			3.21	Swanson 2006
			3.21	Swindle 2008
			3.21	Tessem 2008
		Phosphoethanolamine	3.21	Govindaraju 2000
			3.22	Zektzer 2005
		Glycerophosphocholine	3.21	Van Asten 2008
			3.21	Swindle 2008
			3.22	Stenman 2010, Stenman 2011
			3.24	Swanson 2006
			3.24	Tessem 2008
		Phosphocholine	3.21	Van Asten 2008
			3.21	Swindle 2008
			3.22	Stenman 2010, Stenman2011
			3.23	Swanson 2006
			3.23	Tessem 2008
		Taurine	3.24	Govindaraju 2000
			3.25	Stenman 2010, Stenman 2011
			3.25	Swindle 2008
		Inositol	3.25	Swindle 2008
21	3.13–3.17	Polyamines	3.05–3.15	Stenman 2010, Stenman 2011
			3.10–3.14	Tessem 2008
		Spermine	3.1–3.2	Swindle 2008
			3.14	Van Asten 2008
		Ethanolamine	3.15	Zektzer 2005
22	3.09–3.12	Polyamines	3.05–3.15	Stenman 2010, Stenman 2011
			3.10–3.14	Swanson 2006
		Polyamines (Spermine, Spermidine, Putrescine)	3.11	Tessem 2008
		Spermine	3.09–3.13	Tessem 2008
		Phenylalanine	3.11	Govindaraju 2000
23	3.05–3.08	Lysine	3.05	Swindle 2008
		Polyamine	3.05–3.15	Stenman 2010, Stenman 2011
24	3–3.04	Creatine	3.02	Stenman 2010, Stenman 2011
			3.026	Govindaraju 2000
			3.03	Van Asten 2008
			3.03	Swindle 2008
			3.04	Swanson 2006
		Tyrosine	3.04	Govindaraju 2000
25	2.96–2.99			
26	2.87–2.95			
27	2.8–2.86	PUFA n6 species	2.80	Stenman 2009
		Diallylic protons (Omega 6.20)	2.80	Stenman 2011
		Aspartate	2.80	Govindaraju 2000
		Lipid	2.82	Swindle 2008
28	2.75–2.79			
29	2.69–2.74	Citrate	2.70	Van Asten 2008
			2.70	Dittrich 2012
			2.72	Swanson 2006
30	2.64–2.68	Aspartate	2.65	Govindaraju 2000
		Citrate	2.65	Stenman 2010, Stenman 2011
			2.66	Swindle 2008
			2.67	Van Asten 2008
			2.67	Dittrich 2012
31	2.58–2.63	Citrate	2.62	Tessem 2008
32	2.5–2.57	Citrate	2.51, 2.54	Dittrich 2012
			2.52	Swindle 2008
			2.54	Swanson 2006
			2.55	Stenman 2010, Stenman 2011
33	2.46–2.49	Taurine	2.46	Stenman 2010, Stenman 2011
		Glutamine	2.47	Stenman 2010, Stenman 2011
34	2.39–2.45	Succinate	2.39	Govindaraju 2000
		Glutamine	2.43, 2.45	Govindaraju 2000
35	2.3–2.38	Lipid	2.3	Swindle 2008
			2.33, 2.35	Govindaraju 2000
		Glutamate	2.35	Stenman 2010, Stenman 2011
		Pyruvate	2.36	Govindaraju 2000
36	2.22–2.29	Valine	2.26	Govindaraju 2000
		Lipid	2.27	Giskeødegård 2013
37	2.19–2.2			
38	2.12–2.17	Glutamate	2.12	Govindaraju 2000
			2.15	Stenman 2010, Stenman 2011
		Glutamine	2.13	Govindaraju 2000
			2.14	Stenman 2010, Stenman 2011
39	2.09–2.11	Spermine & Spermidine	2.10	Swanson 2006
		Spermine	2.10	Swindle 2008
		Polyamines (Spermine, Spermidine, Putrescine)	2.10	Tessem 2008
40	2.06–2.08			
41	1.99–2.05	Proline	2.02	Mickiewicz 2014
		Lipid	2.02	Swindle 2008
			2.05	Giskeødegård 2013
		Glutamate	2.04	Govindaraju 2000
			2.05	Stenman 2010, Stenman 2011
42	1.91–1.96	Acetate	1.90	Govindaraju 2000
43	1.82–1.9			
44	1.74–1.8	Polyamines (Spermine, Spermidine, Putrescine) Spermine	1.78 1.78 1.8	Swanson 2006 Tessem 2008 Swindle 2008
45	1.65–1.73	Lysine	1.72	Swindle 2008
46	1.58–1.61	Lipid	1.60	Giskeødegård 2013
			1.6	Swindle 2008
47	1.51–1.56			
48	1.45–1.48	Alanine	1.47	Van Asten 2008
			1.47	Govindaraju 2000
			1.47	Swindle 2008
			1.48	Stenman 2010, Stenman 2011
			1.49	Swanson 2006
			1.49	Tessem 2008
49	1.4–1.44	Lysine	1.44	Swindle 2008
50	1.35–1.39			
51	1.27–1.34	Lactate	1.30	Swindle 2008
			1.31	Govindaraju 2000
			1.33	Van Asten 2008
			1.33	Stenman 2010, Stenman 2011
			1.33	Tessem 2008
			1.34	Swanson 2006
		Threonine	1.31	Govindaraju 2000
			1.31	Swindle 2008
		Lipid	1.33	Swindle 2008
52	1.17–1.26			
53	1–1.06	Valine	1.03	Govindaraju 2000
			1.03	Swindle 2008
54	0.97–0.99	(Iso)Leucine	0.97	Swindle 2008
		Valine	0.98	Govindaraju 2000
55	0.93–0.96			
56	0.77–0.92	Lipid	0.9	Swindle 2008
57	0.68–0.74			
58	0.51–0.53			

Abbreviations: ppm = parts per million.

## Appendix C. Tables of all Significant Results

**Table A3:** Principal components that were significantly different between groups (ANOVA).

Groups	Principal Component	*p*
Gr1 & Gr2	PC11	0.037
	Gr1 & 2 PC11	0.037
Gr1 & Gr3	PC 1	0.021
	Gr1 & 3 PC1	0.011
Gr2 & Gr3	Gr2 & 3 PC6	0.033

Abbreviations: ANOVA = analysis of variance, Gr = group, *p* = *p*-value, PC = principal component.

**Table A4:** Spectral regions with significantly different peak intensities between groups (matched-pair analysis).

Groups	Region	*p*
Gr1 & Gr2	R17	0.021
	R18	0.003
	R20	0.029
	R23	0.018
	R27	0.026
	R40	0.044
	R49	0.040
Gr1 & Gr3	R17	0.029
	R18	0.013
	R24	0.036
	R35	0.009
Gr2 & Gr3	R23	0.005
	R36	0.023
	R44	0.029
	R46	0.016
	R52	0.032

Abbreviations: Gr = group, *p* = *p*-value, PC = principal component, R = region.

**Table A5:** Spectral regions with significantly different peak intensities between samples of patients with highest Gleason scores in the study period of 3 + 3 = 6 and 3 + 4 = 7.

Gleason Scores	Region	*p*
3 + 3 = 6 & 3 + 4 = 7	R23	0.048
	R27	0.021
	R28	0.013

Abbreviations: Gr = group, *p* = *p*-value, R = region.

**Table A6:** Significant linear correlations between spectral peak intensities or principal components and the Vol.%Epi.

Groups	Region/PC	*p*	r
All	R23	0.0399	0.2976
	R30	0.0008	0.4670
Gr1	R1	0.0015	0.7251
	R8	0.0219	0.5675
	R16	0.0095	0.6257
	P11	0.0414	−0.5146
	Gr1 PC10	0.0304	−0.5411
Gr2	R30	0.0487	0.4999
Gr3	R54	0.0006	−0.7610

Abbreviations: Gr = group, PC = principal component, R = region, r = r value of correlation.

**Table A7:** Significant linear correlations between spectral peak intensities or principal components and the PSAd.

Groups	Region/PC	*p*	r
All	R9	0.0253	−0.3227
	R50	0.0213	−0.3317
	PC9	0.0158	−0.3466
Gr1	R9	0.0232	−0.5631
	Gr1 PC8	0.0195	0.5762
Gr2	R9	0.0487	−0.4999
Gr3	R3	0.0326	0.5354
	R4	0.0260	0.5539
	R53	0.0047	0.6682
	R58	0.0002	0.7984
	PC4	0.0238	−0.5608
	Gr3 PC3	0.0061	0.6532

Abbreviations: Gr = group, *p* = *p*-value, PC = principal component, PSAd = PSA density, R = region, r = r value of correlation.
